# RAR/RXR and PPAR/RXR Signaling in Spinal Cord Injury

**DOI:** 10.1155/2007/29275

**Published:** 2007-08-02

**Authors:** Sabien van Neerven, Jörg Mey

**Affiliations:** ^1^Institute of Biology II, RWTH Aachen University, 52056 Aachen, Germany; ^2^EURON Graduate School of Neuroscience, 6229 Maastricht, The Netherlands

## Abstract

The retinoid acid receptors (RAR) and peroxisome proliferator-activated receptors (PPAR) have been implicated in the regulation of inflammatory reactions. Both receptor families contain ligand-activated transcription factors which form heterodimers with retinoid X receptors (RXR). We review data that imply RAR/RXR and PPAR/RXR pathways in physiological reactions after spinal cord injury. Experiments show how RAR signaling may improve axonal regeneration and modulate reactions of glia cells. While anti-inflammatory properties of PPAR are well documented in the periphery, their possible roles in the central nervous system have only recently become evident. Due to its anti-inflammatory function this transcription factor family promises to be a useful target after spinal cord or brain lesions.

## 1. INTRODUCTION

During the last decade much progress has been made disentangling the physiological responses after central nervous system (CNS) injury. Yet, there is still no effective treatment available to prevent the devastating effects that result from major CNS lesions. Although neurite sprouting and axonal growth is observed after spinal cord injury (SCI), successful regeneration beyond the lesion-induced scar does not occur. In contrast, axonal regeneration with functional recovery is possible in the peripheral nervous system (PNS). The different outcomes of CNS and PNS lesions are largely due to differences in the cellular and molecular signals that neurons encounter in these environments. Three important problems after CNS injury are (1) development of a growth inhibitory glial scar, (2) secondary neuronal and glial degeneration as a delayed consequence of the lesion, and (3) the failure of axonal regeneration in white matter tracts. Responding to these problems, most attention has been directed either to overcome the inhibitory barrier of the glial scar or to promote the growth of axon collaterals and thus compensate for permanently severed connections [1–3]. A subsidiary approach is to modify inflammatory reactions in order to limit the secondary degeneration. Synthetic corticosteroids, e.g., methylprednisolone, are the only pharmacological tools currently in clinical use [4]. In this review, we will discuss two nuclear receptor families that have recently been discovered as possible therapeutic targets for the treatment of SCI and brain damage. Retinoic acid receptor (RAR) signaling may improve axonal regeneration, influence glial differentiation, and modulate inflammatory reactions [5]. Peroxisome proliferator-activated receptors (PPAR) have a major function in anti-inflammatory processes [6]. Both receptor classes, RAR and PPAR, heterodimerize with retinoid X receptors (RXR) to form active transcription factors.

## 2. ENDOGENOUS RAR/RXR AND PPAR/RXR ACTIVITY IN THE INJURED NERVOUS SYSTEM

### 2.1. RAR/RXR signal transduction

The RAR/RXR transcription factor complex is activated by ligand binding, its natural ligand being all-*trans* retinoic acid (all-*trans* RA). Retinoids are obtained from the diet in the form of vitamin A (retinol and retinal), retinyl esters, or *β*-carotene. Following cellular uptake of all-*trans* retinol from the plasma, the intracellular synthesis of retinoic acid occurs in two steps. (1) Retinol is oxidized to retinal, predominantly involving the alcohol dehydrogenases ADH-1, -3, and -4. (2) The critical step is the subsequent oxidation of retinal to retinoic acid by retinaldehyde dehydrogenases (RALDH) [7]. Retinoic acid is released in a paracrine or autocrine fashion. The fact that RA activates nuclear transcription factors was discovered in 1987 [8, 9]. Retinoid receptors belong to the same superfamily as PPAR, thyroid hormone receptors (TR), and steroid receptors. They can be grouped into two families, the RARs and the RXRs, each consisting of three isoforms encoded by separate genes: RAR *α*, RAR*β*, RAR *γ* (also: NR1B1-3) and RXR*α*, RXR*β*, RXR*γ* (NR2B1-3). All-*trans* RA and 9-*cis* RA bind to the RAR family, whereas only 9-*cis* RA is a high-affinity ligand for the RXR [7, 10]. The active transcription factor complex consists of an RAR/RXR heterodimer, ligand, and coactivators. It interacts with retinoic acid response elements (RARE) in the promoters of target genes. About 500 genes have been suggested to be regulated by RAR/RXR signaling, however a much lower number was experimentally shown to be activated via the classical RARE driven pathway. Proven target genes include enzymes, transcription factors, cytokines, and cytokine receptors [5, 11]. In addition, many cases of gene suppression and nongenomic modes of action of RA and its receptors have been described [7].

### 2.2. RAR/RXR signaling after spinal cord injury

**Figure 1:**
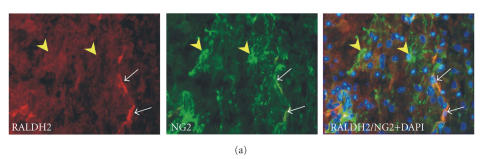

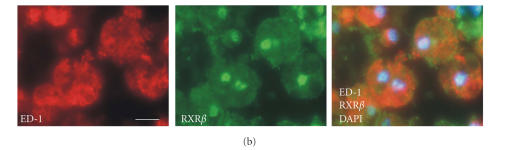

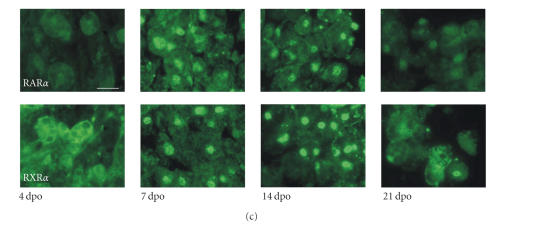
Retinoic acid synthesis and nuclear translocation of RAR/RXR after spinal cord injury. (a) Double immunostaining of rat spinal cord sections shows RALDH-2 immunoreactivity (red) in a subpopulation of NG2-positive glia (green) 7 days after contusion injury. RALDH2/NG2 expressing cells were only detected in the vicinity of the lesion site, many NG2 cells do not contain the RA-synthesizing enzyme (yellow arrow heads). White arrows point to DAPI-stained cell nuclei of RALDH2/NG2 cells in superimposed photographs. (b) At 7 dpo, ED1-positive macrophages at the lesion site express RXR*β* in their cell nuclei. (c) SCI-induced transient translocation of RAR*β* and RXR*α* from the cytosol into the nuclei of activated macrophages/microglia. All scale bars are 20*μ* *m* (sources: [12, 14]).

The discovery that enzyme activity of a retinaldehyde dehydrogenase increased after spinal cord contusion injury was the first direct evidence that retinoids play a role in the physiological responses to SCI. Contusion injury caused a significant increase in RALDH2 enzyme activity, which peaked 8–14 days following the lesion [12]. While in the noninjured rat spinal cord RALDH2 is only present in the meninges, oligodendrocytes and in pericytes, around the lesion site, its immunoreactivity also appeared in a population of NG2-positive glia cells [13] (Figure 1(a)). NG2 is a chondroitin sulfate proteoglycan, expressed in cells that have been described as oligodendrocyte precursor cells, synantocytes or polydendrocytes. These cells respond to injury with increased production of NG2 and a subpopulation of them, close to the lesion site, appears to be involved in the local production of RA. Alternatively or additionally, RA-synthesizing cells migrate from the adjacent arachnoid membrane and from blood vessels toward the site of injury [13]. While only minor changes in the quantitative expression of retinoid receptors were observed after SCI, their cellular distribution changed remarkably (Figures 1(b), 1(c)). In the noninjured tissue, retinoid receptors were found in the cytosol of motorneurons and glia, but close to the injury site macrophages and surviving neurons displayed a nuclear localization of RAR*α*, RXR*α*, and RXR*β* [14]. In the context of locally rising RA synthesis, the observation that retinoid receptors translocate into the cell nuclei indicates that neurons, glia, and macrophages are targets of RA signaling after SCI. This interpretation is consistent with data on spinal cord development, where RA has been shown to be a regulator of cell differentiation [15–18].

### 2.3. RAR/RXR signaling after peripheral nerve injury

RA signaling is implicated in the differentiation of neurons whose axons grow in the PNS, [19–22] and again in neurite regeneration [23, 24]. Several mediators of the RA signaling pathway have been shown to be induced after peripheral nerve injury in the rat. Experimental lesion of the sciatic nerve induced expression of the cellular retinol binding protein-I (CRBP-I) [25], which is implicated in retinoid metabolism [26]. Similarly, transcript and protein concentrations of the cellular retinoic acid binding protein-II (CRABP-II) increased strongly [25]. CRABP-II is probably involved in the intracellular transport of RA to the cell nucleus [27]. As after SCI, the RA-synthesizing enzyme RALDH2 was present in the injured nerve. After sciatic nerve injury in transgenic reporter mice, local activation of RARE was detectable in the regenerating nerve, indicating that RA-dependent gene expression is induced during peripheral nerve regeneration [25]. The expression of retinoid receptors also changed significantly. Following sciatic nerve crush, mRNA of all RARs and of RXR*α* was increased, and at 4, 7, and 14 days after the injury protein levels of RAR*α*, RAR*β*, and RXR*α* were augmented [28]. To study Wallerian degeneration in the absence of axonal regeneration, the distal nerve segment can be cut off and sutured to the peroneus muscle. Under these conditions RAR*α* and RAR*β* were upregulated in the degenerating nerve.

Cell culture experiments indicated that RAR*β* is required for RA-induced axonal regeneration [24]. Consequently, experiments with RAR*β*-deficient mice corroborated this interpretation because there were significantly less GAP43-positive, regenerating axons after sciatic nerve crush in the knockout animals [29]. Since RALDH2 [23] and RAR*β* [30] are induced by NGF, RA appears to act downstream of this neurotrophin. RA/RAR*β* signaling seemed to be necessary for the neurotrophic activity of NGF [23]. In addition, neurotrophin-independent RA/RAR*β* activity contributes to axon outgrowth [29]. Double labeling with cell type specific markers in the sciatic nerve demonstrated expression of retinoid receptors mostly in macrophages and Schwann cells. In cultured primary Schwann cells, RA downregulated CNTF [31] and raised expression of ErbB3, a neuregulin receptor that is necessary for normal myelination. Finally, RXR*α* labeling was shown to colocalize with some regenerating axons, indicative of a direct role for RXR*α* in nerve regeneration [28].

### 2.4. PPAR/RXR signal transduction

Peroxisome proliferator-activated receptors belong to the same superfamily as the RAR. The first PPAR was discovered, cloned, and sequenced in 1990, and it was named after its property to be activated by drugs that cause proliferation of peroxisomes in hepatocytes [32]. Until now, three different isoforms of PPAR, encoded by separate genes, have been identified: PPAR*α* (NR1C1), PPAR*β*/*δ* (NUC1, NR1C2), and PPAR*γ* (NR1C3). The different isoforms have similar structural features, however each isoform exhibits its own specific tissue expression pattern and distinct physiological functions depending on ligand activation [33, 34]. PPAR also heterodimerize with RXR, then bind as a complex to their response elements (PPRE). These consist of two repeats of the consensus sequence AGGTCA separated by one or two nucleotides (direct repeats DR-1 and DR-2) [35]. Natural ligands of PPAR are fatty acids, prostaglandins, and oxidized fatty acid derivatives. They are also activated by synthetic ligands like the lipid-lowering fibrates and the anti-diabetic glitazones [36]. PPAR, which have been implicated in lipid metabolism, cellular proliferation and inflammatory responses, are widely expressed, for example, in colon, spleen, retina, the cardiovascular system, liver, skeletal muscles, and in adipose tissue. Their expression by monocytes, dendritic cells, endothelial cells, megakaryocytes, and lymphocytes may be related to immune functions [34, 36]. PPAR*γ* can also influence gene expression independently of PPRE. The activity of a number of transcription factors, for example, NF*κ*B, AP-1, and STAT-1, are inhibited by PPAR*γ* via direct interaction or by competition for limiting supplies of coactivators [6].

### 2.5. PPAR/RXR signaling after spinal cord injury

**Figure 2:**
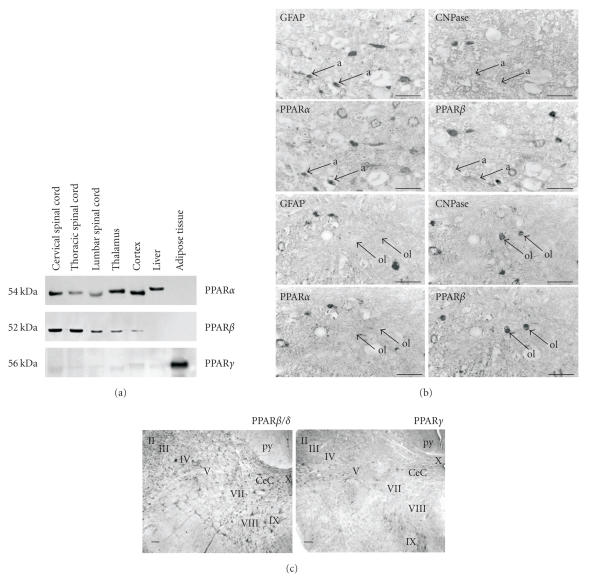
Localization of PPAR*α* and PPAR*β*/*δ* in astrocytes and oligodendrocytes in the spinal cord. (a) Western blotting shows PPAR*α* and PPAR*β* but not PPAR*γ* in spinal cord, telencephalon, and diencephalon. Identical expression patterns were detected with RT-PCR. (b) Detection of PPAR immunoreactive cells in the white matter of rat spinal cord. GFAP-positive/CNPase-negative astrocytes are immunoreactive for PPAR*α* (marked a, upper four panels) while GFAP-negative/CNPase-positive oligodendrocytes express PPAR*β* (marked ol, lower panels) (scale bars: 25*μ* *m*, source: [38]). (c) Distribution of PPAR*β*/*δ* and PPAR*γ* immunoreactive cells in the spinal cord of the adult rat (cervical level, coronal sections, scale bar: 100*μ* *m*, source: [41]).

PPAR are expressed in the developing and adult CNS. PPAR*α* and PPAR*β*/*δ*, but not PPAR*γ* were found in cervical, thoracic, and lumbar segments of the adult spinal cord, in the thalamus and cerebral cortex [37] (Figure 2). Immunohistochemical staining showed that PPAR*β*/*δ* is the main isoform present in neuronal cell bodies of the spinal cord gray matter. Both receptors, PPAR*α* and PPAR*β*/*δ*, were concentrated in cell nuclei. In the white matter PPAR*α* appeared particularly strong in PPAR*β*/*δ*-negative astrocytes, whereas oligodendrocytes expressed only PPAR*β*/*δ* [38]. PPAR*β*/*δ* is a factor in neuronal differentiation, and functions of this receptor in various aspects of neural physiology have been suggested [38, 39]. While in two studies PPAR*γ* could not be detected in brain and spinal cord [37, 38], this receptor is expressed in microglial primary cultures [40] and may be upregulated after injury. In a different report, all PPAR and RXR were found immunohistochemically throughout the adult rat CNS [41].

Endogenous PPAR ligands may play a role in modulating the inflammatory response after SCI, possibly preventing the expansion of the initial damage. Genovese and coworkers [42] examined the effects of endogenous PPAR*α* ligands in an experimental model of spinal cord trauma. SCI was induced in wild-type and in PPAR*α*-deficient (−/−) mice. The injury resulted in severe trauma characterized by edema, loss of myelin, neutrophil infiltration, apoptosis, and increased production of TNF*α*. Compared to wild-type animals, all these parameters were augmented in PPAR*α*−/− mice. The absence of PPAR*α* also interfered with recovery of limb function [42]. Studies using animal models of chronic pain indicate that PPAR*α* is involved in the neural processing of pain [43, 44].

## 3. REGULATION OF INFLAMMATORY PROCESSES BY RAR/RXR SIGNALING IN THE SPINAL CORD

Although the inflammatory response limits the effects of a pathogenic insult, it is also responsible for most of the secondary damage incurred after spinal cord injury. The regulation of these events is therefore of primary therapeutic concern. Both pathways, RAR/RXR and PPAR/RXR signaling are implicated.

### 3.1. Inflammatory reactions in the spinal cord

The physiological events following spinal cord injury can be differentiated into an acute phase (the first 24hrs), followed by a subacute phase (24–72hrs), and a late phase (3–90 days). Events after this time are considered chronic. CNS injury directly leads to release of inflammatory signals resulting in the production of vasoactive mediators and chemotactic factors. Primary damage consists mainly of severed axons, neuronal and oligodendrocyte cell death. Expansion of the initial damage is then caused by a disruption of the blood supply and an extended inflammatory reaction. Depending on the type of injury, the blood-brain barrier is disrupted. This event determines the extent to which blood-derived cells participate in the inflammatory process [45, 46]. When the blood-brain barrier breaks down, the release of cytokines and chemokines leads to a massive recruitment of inflammatory cells from the periphery, including hematogenous neutrophils and perivascular/meningeal macrophages. These cells contribute to tissue damage by the production of proteolytic enzymes and reactive oxygen species. Microglia and macrophages phagocytose dead or injured neurons and glia, and are responsible for the clearance of cellular debris [45].

Microglia, perivascular macrophages, and activated astrocytes are the most important local sources of cytokines. Astrocytes are further involved in the formation of a glial scar, thereby insulating the healthy tissue from uncontrollable processes in the damaged area. Acute inflammation can develop into a chronic process when feedback mechanisms fail to inhibit amplification of the inflammatory response. Chronic inflammation leads to a continuous influx of neutrophils, macrophages, lymphocytes, and eosinophils from the circulation, causing more tissue destruction and scarring.

### 3.2. RAR/RXR signaling is involved in inflammation

Before the molecular mechanisms of the retinoid signaling pathway were understood, anti-inflammatory properties of RA had already been described. In 1983, a study showed that oral administration of retinoids affected dermal inflammatory cells and reduced elevated skin temperature [47]. Retinoic acid has beneficial effects in diseases with an inflammatory-based pathology including asthma, arthritis, and atherosclerosis [48, 49]. A number of cell culture studies support the hypothesis that RA reduces inflammatory activation of monocytes, macrophages, myeloma cells, and polymorphonuclear cells (neutrophil granulocytes). Signals that were found to be downregulated by retinoids include IL-1*α*, IL-1*β*, IL-6, TNF*α*, IL-8, prostaglandin E_2_, production of reactive oxygen species, and release of lysosomal enzymes. A table with these effects and references is presented in [5]. In contrast to a majority of findings that indicate an anti-inflammatory role of RAR/RXR signaling, it was also reported that 9-*cis* RA induced secretion of MCP-1 and thereby stimulated monocyte migration [50]. In combination with T-cell stimulating agents, all-*trans* RA, 9-*cis* RA, and an RAR agonist, TTNPB, indirectly increased proliferation of human T lymphocytes and IL-2 secretion [51].

One of the major transcription factors responsible for the regulation of inflammatory cytokines is NF*κ*B. It was shown that RXR inhibits NF*κ*B-dependent gene expression (IL-12) [52]. In the experiments, interference of RXR with DNA binding of NF*κ*B required the presence of the RXR ligand 9-*cis* RA. The negative RXR/NF*κ*B interaction appears to be mutual because increasing levels of transfection with NF*κ*B subunits also abrogated expression of a retinoid reporter construct [52].

### 3.3. The role of RAR/RXR in inflammatory reactions after CNS injury

**Figure 3:**
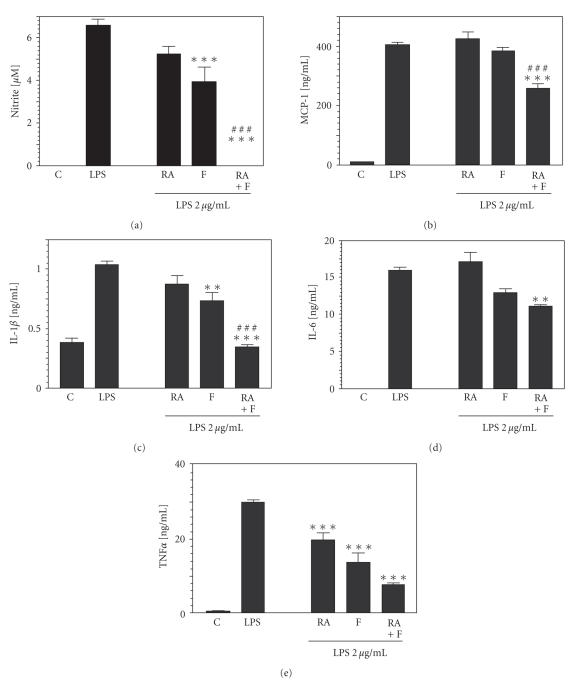
Anti-inflammatory effects of PPAR and RAR agonists in astrocytes. Astrocyte primary cultures from cortices of newborn mice were treated with 9-*cis* RA (RA) and/or the PPAR*α* agonist fenofibrate (F) for 1hour and then stimulated with 2*μ*g/mL LPS for 24 hours. (a) Nitrite production as an indicator of NOS activity was reduced by 1*μ* *M* RA or 100*μ* *M* F, and completely suppressed with RA plus F. Pretreatment of RA and/or F also decreased the release of (b) the chemokine MCP-1 (RA: 2*μ* *M*, F: 200*μ* *M*), and cytokines (c) IL-1*β* (RA: 1*μ* *M*, F: 100*μ* *M*), (d) IL-6 (RA: 2*μ* *M*, F: 50*μ* *M*), and (e) TNF*α* (RA: 1*μ* *M*, F: 100*μ* *M*) in response to LPS. Cytokine production was measured with ELISA. Experiments with microglia cultures revealed similar effects, except for MCP-1, whose production was stimulated by 9-cis RA in microglia. Error bars indicate SEM, asterisks indicate significant differences compared to the LPS condition (sources: [54, 78]).

Although retinoic acid has so far not been used to modify inflammatory reactions in the CNS in vivo, cell culture experiments demonstrate anti-inflammatory effects on microglia and astrocytes [5]. Primary cultures of microglia and astrocytes are most frequently prepared from cerebral cortex of perinatal rats and mice. One can expose these cultures to LPS to simulate a bacterial infection, or treat them with *β*-amyloid peptide (A*β*) to mimic the immunogenic stimulus of Alzheimer's disease. In this kind of experiment, RA treatment suppressed the induction of TNF*α* and iNOS. Effects correlated with enhanced expression of RAR*β*, TGF*β*, and inhibition of NF*κ*B nuclear translocation [53]. Xu and Drew demonstrated that 9-*cis* RA suppressed LPS-induced production of NO as well as of proinflammatory cytokines TNF*α*, IL-1*β*, and IL-12p40 in primary mouse microglia, while IL-6 secretion and MCP-1 production were not significantly affected (Figure 3) [54]. In astrocytes from rat brain and in C6 astroglioma cells, 9-*cis* RA and all-*trans* RA inhibited IFN*γ*-induced inflammatory responses [55].

Matrix metalloproteinases (MMP) are involved in the breakdown of the extracellular matrix and other proteins. These proteases are products of leukocytes and endothelial cells and are released in response to various cytokines and growth factors. Increased proteolytic activity of MMP can lead to disruption of the blood-brain barrier and escalate the inflammatory response [56, 57]. Retinoic acid has negative effects on secretion and expression of these enzymes. It reduced mRNA, protein synthesis, and secretion of MMP-9 (gelatinase B) in lymphocytes from patients with chronic B-lymphocytic leukemia and in human monocytes [58]. Other metalloproteinases including MMP-1 and MMP-13 appeared to be influenced by RA as well [59].

## 4. REGULATION OF INFLAMMATORY PROCESSES BY PPAR/RXR SIGNALING IN THE SPINAL CORD

### 4.1. PPAR/RXR signaling counteracts inflammatory processes

For the PPAR family, anti-inflammatory effects are well documented. One mechanism involves direct interaction of PPAR with proinflammatory transcription factors, most importantly NF*κ*B and AP-1, and the subsequent reduction of gene transcription. This has been observed in vitro in human vascular endothelial cells [60], human aorta smooth muscle cells [61], in C2C12 skeletal muscle cells (in a model for insulin resistance in type 2 diabetes) [62], and in fibroblasts from rheumatoid arthritis patients [63]. PPAR knockout studies supplement and confirm these results. One of the first reports indicating that PPAR*α* is involved in attenuating inflammation demonstrated that the eicosanoid LTB_4_ binds and activates PPAR*α*. Subsequently, PPAR*α*-deficient mice were shown to have a prolonged inflammatory response when challenged with LTB_4_ or arachidonic acid [64].

An interaction of PPAR*α* with NF*κ*B is indicated by studies of inflammatory cytokine production in aging. In aged mice, NF*κ*B becomes constitutively active in many tissues due to oxidative stress, which eventually leads to the production of cytokines. Administration of PPAR*α* activators was found to restore the cellular redox balance, to suppress the constitutive activation of NF*κ*B and to eliminate the spontaneous production of IL-6 and IL-12 [65, 66]. Other examples where PPAR*α*−/− mice suffer from more severe inflammatory reactions are carrageenan-induced hypersensitivity [67], airway inflammation [68], and experimental colitis [69]. On the molecular level, these investigations demonstrated Fas-ligand, IL-1*β*, TNF*α*, keratinocyte-derived chemokine, MIP-2, MCP-1, ICAM-1, and enzyme activities of MMP-9, myeloperoxidase, and iNOS to be influenced by PPAR*α* signaling [67, 69, 70].

PPAR*γ*-deficient mice die in utero, but heterozygotic PPAR*γ*+/− mice with 50% expression of the receptor survive and can be studied. In an investigation of PPAR*γ* functions after intestinal and gastric ischemia/reperfusion injury, PPAR*γ*+/− mice displayed more severe lesions of the gastric intestinal mucosa. Treatment with the PPAR*γ* ligand BRL-49653 limited the damage of intestinal injury and resulted in downregulation of cell adhesion molecules and proinflammatory cytokines in the intestine and stomach [71, 72]. As mentioned before, NF*κ*B and inflammatory mediators are raised during the aging process. While this is accompanied by reduced PPAR*γ* levels, the PPAR*γ* ligand 2,4-TZD was shown to reduce age-related oxidative stress, the translocation of NF*κ*B p65 subunit to the nucleus and NF*κ*B-regulated transcription of iNOS, COX-2, IL-1*β*, IL-6, and the cell-adhesion molecule VCAM-1 [73]. Another PPAR*γ* agonist, rosiglitazone, relieved renal injury in a nephrotoxicity model, also acting via inhibiton of NF*κ*B [74]. It seems therefore that both receptors PPAR*α* and PPAR*γ* play a general role in modulating the inflammatory response in a wide variety of tissues.

### 4.2. Effect of PPAR activation on macrophages, microglia, and astrocytes

Given that PPAR agonists influence peripheral macrophages [75], it was to be expected that they act on brain macrophages after disruption of the blood-brain barrier. PPAR*α* is already present in undifferentiated monocytes, while PPAR*γ* expression is induced during differentiation [76, 77] and upregulated in the course of macrophage activation [78–82]. A number of cell culture studies with macrophages and microglia demonstrate that agonists of PPAR*α* and PPAR*γ* elicit the same anti-inflammatory effects that we have discussed above. When cells were activated with bacterial antigens or A*β*, the PPAR ligands typically prevented or reduced the inflammatory response. This effect was observed on the following levels: (1) activity of transcription factors NF*κ*B, AP-1, and STAT-1, (2) secretion of proinflammatory cytokines and chemokines, (3) enzyme activities of COX-2, iNOS, and MMP-9, and (4) the formation of reactive oxigen radicals. A synopsis of these results is presented in Table 1.

Such effects were observed with natural ligands, for example, prostaglandins, with artificial PPAR activators, for example, antidiabetic thiadiazolidinones, and with nonsteroid anti-inflammatory drugs [83]. A very efficient endogenous agonist of PPAR*γ* is the prostaglandin 15d-PGJ2 [70]. However, this drug also interferes with the transcriptional activity of NF*κ*B independently of PPAR*γ* activation because overexpression of PPAR*γ* or an antagonist of PPAR*γ* did not alter the 15d-PGJ2 effect on LPS/IFN*γ*-dependent inflammatory reaction. It was concluded that 15d-PGJ2 inhibits the inflammatory response by directly regulating the NF*κ*B and PI3K-Akt pathways [79, 84].

With respect to microglia effects on nerve cells, PPAR activation with synthetic ligands can be neuroprotective. When cortical neurons were exposed to the cell-free supernatant from activated microglia, neurons survived better when microglia had also been incubated with PPAR*γ* agonists [85]. Some experiments in vivo also support this concept. Oral administration of pioglitazone or the nonsteroidal anti-inflammatory drug ibuprofen, which is also a ligand for PPAR*γ*, reduced the number of activated microglia and reactive astrocytes in the hippocampus and cerebral cortex. This was observed in 10-month-old APPV717I mice, an inflammation model for Alzheimer's disease [83]. Pioglitazone protected mice also from MPTP-induced dopaminergic neuronal cell loss and mitigated inflammatory response by microglia in an animal model of Parkinson's disease [86].

As mentioned before, astrocytes participate in the secretion of cytokines and form a glial scar around the damaged tissue. Since the production of microglia primary cultures also involves isolation of astrocytes, it was a straightforward approach to test PPAR agonists on this type of glia as well (Figure 3) [87–89]. Results, which were generally in line with the anti-inflammatory effects on microglia, are also included in Table 1.

## 5. APPLICATION OF RETINOIDS AND PPAR LIGANDS AFTER SPINAL CORD AND PERIPHERAL NERVE INJURY

Retinoic acid is a well-characterized morphogenetic factor in embryonic development. Stem cells and several cell lines can be induced with RA to differentiate to a neuronal phenotype. A number of experiments with developing sympathetic neurons and with sensory neurons from dorsal root ganglia revealed that RA has neurotrophic effects or induces neurite growth. The question arises whether these properties can be exploited to support axonal regeneration [5, 90].

### 5.1. Retinoic acid-induced axonal growth in vitro and in vivo

While the most convincing demonstration that RA can act as an axon-guiding chemoattractant was recently published using the snail *Lymnaea stagnalis* [91], a number of earlier studies indicated that retinoids may fulfill similar functions in vertebrates. Neurites extending from chick neural tube cells responded to a gradient of RA [92]. Positive effects on axon outgrowth were also reported using explants from murine embryonic spinal cord [93], embryonic cerebellum [94], and amphibian spinal cord [95]. In a chick retinal explant assay, RA enhanced neurite outgrowth under the condition that the neurotrophin BDNF was subsequently added in vitro [96]. RA supported survival and morphological differentiation of cultured rat spinal cord neurons and increased neurite density. In this context, astrocytes were implicated as regulators of local RA concentration [16, 17].

**Figure 4:**
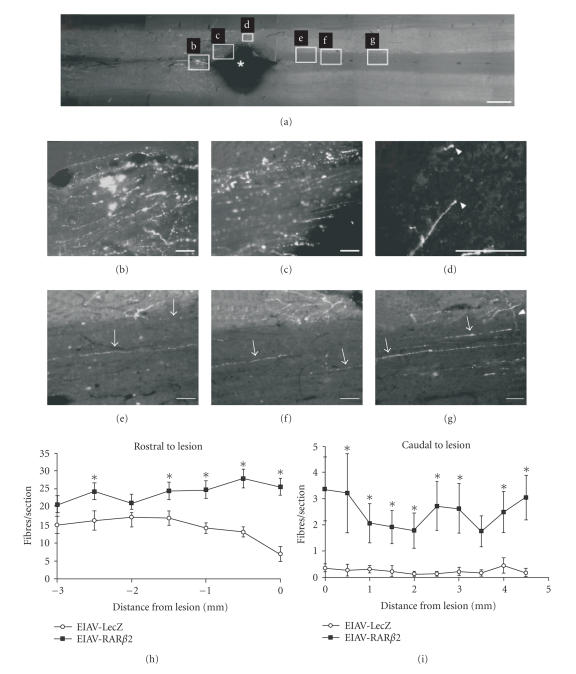
Transfection of RAR*β* induces regeneration of corticospinal axons in vivo. (a) Longitudinal sections of the adult rat spinal cord were traced with BDA to determine fibre regeneration in the corticospinal tract; overview of the lesion site in an animal injected with the RAR*β*2 expressing construct (EIAV-RAR*β*2, Rostral: left). (b) Animals with RAR*β*2-transfected cells displayed less fiber degeneration than control rats. (c) Labeled axons were detected up to the edge of the lesion, (d) at the distal edge of the lesion, and (e–g) at various distances caudal to the lesion. Growth cone-like endings were detected at the tips of some axons (white arrowheads in d). Axons appear to send collaterals (white arrowhead in g) from white matter to grey matter. (Scale bars in (a): 1mm, in (b–g): 100*μ* *m*.) (h–i) Quantification of BDA-labeled fibres in the corticospinal tract after spinal cord lesion. EIAV-RAR*β*2-treated animals displayed increased fiber numbers rostral and caudal to the lesion compared to control animals (*P* < .05, two-way ANOVA) (source: [100]).

Corcoran, Maden, and coworkers suggested that the loss of regeneration in the adult mammalian CNS is related to developmental changes in retinoid signaling. They observed that the RAR*β*2 receptor, which is present when neurite outgrowth occurs from embryonic spinal cord explants, was not detectable in the adult spinal cord [97]. A supporting result was that specifically RAR*β* mediated the induction of neurite outgrowth from subpopulations of sensory neurons [24]. In addition to the inhibitory glial environment [1], intrinsic changes in CNS neurons have been assumed to be responsible for the loss of regenerative ability during CNS development [98]. The declining expression of RAR*β*2 may be one of those changes. To test this hypothesis, the RAR*β*2 gene was transfected into spinal cord explants with a lentiviral vector. This treatment allowed the outgrowth of many neurites, implying the involvement of RAR*β*2 in axonal regeneration [97]. Consequently, to demonstrate that a renewed expression of the appropriate receptor mechanism might induce axonal regeneration into the spinal cord in vivo, Wong and others [99] transfected RAR*β*2 in adult rats. They then analyzed regeneration of sensory axons into the spinal cord and performed behavioral experiments. Following complete dorsal root transections by means of a crush injury, axons regenerated across the dorsal root entry zone into the CNS. Both myelinated and nonmyelinated fibers were found to have grown in the spinal cord where they projected into the gray matter, formed functional connections and improved sensorimotor recovery of the animals [99]. In another study, artificially induced RAR*β*2 expression also supported regeneration of descending corticospinal tract fibers after midcervical spinal cord injury (Figure 4). Again, the induced regeneration of fibers was accompanied by improved sensory and locomotor behavior [100]. These data suggest not only that RAR*β* is involved in activating a physiological program of regeneration but also that retinoic acid signaling may be used as a therapeutic tool in spinal cord injury.

### 5.2. Manipulation of RAR/RXR signaling in the peripheral nervous system

As mentioned above, it has been known for some time that retinoids act as neurotrophic factors for sympathetic and sensory neurons of the peripheral nervous system [20–24]. However, very few studies have addressed the role of RA in peripheral nerve regeneration in vivo. Taha and coworkers tested whether local RA injections improved morphological and functional regeneration of tibial nerves undergoing anastomosis. They showed that animals injected locally with RA exhibited increased axonal density when compared to vehicle-treated animals [101]. Several cell culture experiments suggested that RA effects may involve an upregulation of neurotrophin receptors, and molecular studies indicate mutually synergistic influences of the RA- and neurotrophin-signaling pathways. Nerve growth factor induced the RA-synthesizing enzyme RALDH in dorsal root ganglia [23] and activated the RAR*β* promoter [30]. RA, on the other hand, caused expression of various neurotrophin receptors (see [5, 90] for review). That synergistic interactions between RA and neurotrophins take place in vivo was revealed in a study using a mouse model for diabetes [102]. Treatment of the animals with all-*trans* RA via subcutaneous injection in a dose of 20mg/kg was able to prevent the NGF depletion normally seen in diabetic mice. Microscopic analysis subsequently showed that diabetes-associated loss of Schwann cells, of myelinated, and of nonmyelinated axons was strongly reduced by RA. This beneficial effect was also measurable in behavioral tests of sensory-motor functions [102].

### 5.3. Retinoids and PPAR ligands in pain signaling

In a different line of research about spinal cord sensitization, some contrasting results were obtained recently. To investigate whether retinoids might be involved in the processing of nociceptive information, especially in situations of hyperalgesia due to inflammation, rats were given 10–15mg/kg all-*trans* RA orally for 4 days. Electrophysiological recordings revealed that treated animals had decreased thresholds to mechanical and electrical stimulation of the paws and increased cutaneous receptive fields. This increased responsiveness caused by RA was similar to hyperalgesia induced by intraplantar administration of carrageenan (which leads to local inflammation) [103]. On the molecular level, RA induced changes of gene expression in the spinal cord, for instance, an increase of COX-2 and IL-1. Inhibition of these pathways with IL-1 receptor antagonist and the COX-inhibitor dexketoprofen reduced responses to mechanical or thermal stimulation when those had been sensitized with RA [104]. In these studies, it was concluded that all-*trans* RA induced changes in the spinal cord that are similar to inflammation. At this point, it is difficult to reconcile this conclusion with the anti-inflammatory properties when RA was combined with stimuli of bacterial infection or A*β* deposition.

In contrast to RA, administration of the PPAR*α* agonists GW7647, Wy14643, perfluorooctanoic acid, and PEA reduced nocifensive behaviors in various animal models of hyperalgesia, implicating a mitigating role of PPAR in the regulation of pain signaling [43, 44]. GW7647 and PEA also prevented firing of spinal cord nociceptive neurons in rats after peripheral exposure to formalin [44].

### 5.4. Use of PPAR ligands as anti-inflammatory treatment after spinal cord injury

The knowledge about anti-inflammatory properties of PPAR ligands obtained in cell cultures soon prompted animal experiments. In these, PPAR*α* ligands were indeed shown to exert neuroprotective effects after traumatic brain injury [105]. Recently, it was discovered that synthetic PPAR*γ* ligands rosiglitazone and pioglitazone are also neuroprotective by reducing the inflammation in the spinal cord. Treatment of rats directly after SCI resulted in a decreased lesion site, better survival of motor neurons, sparing of myelin, reduced astrogliosis, and less microglial activation. These features were accompanied by enhanced functional motor recovery and reduced hyperalgesia [106]. Investigation of gene expression revealed that pioglitazone lowered levels of transcription factors IRF-1, NF*κ*B, transmembrane proteins Egr-1, ICAM, and cytokine/chemokines IL-1*β*, IL-6, MCP-1, but increased expression of neuroprotective genes and antioxidant enzymes. In a rat model for stroke (middle cerebral artery occlusion), PPAR*γ* ligands were able to improve neurological outcome, decreased infarct size, and reduced inflammation. Fewer microglia/macrophages appeared to be present, and transcript and protein levels of IL-1, COX-2, and iNOS were also lower [107].

## 6. CONCLUSION

The studies discussed here demonstrate that RAR/RXR and PPAR/RXR signaling may be therapeutic targets after spinal cord injury. The main potential of PPAR ligands derives from their anti-inflammatory capacity, which has been corroborated in rodent models of inflammation involving various peripheral organs. After spinal cord injury, inflammatory reactions account for a large proportion of the secondary damage to neurons and oligodendrocytes. In this context, first experiments show that PPAR ligands are neuroprotective. Since PPAR activators are already in use to treat diabetes, clinical studies after stoke or other kinds of CNS damage are to be expected. While retinoids also have anti-inflammatory properties, they exert more direct effects on the nerve tissue. In several populations of neurons, axonal regeneration can be induced or supported by the activation of RAR/RXR signaling. Since systemic application of retinoic acid influences neural physiology within the CNS, therapeutic possibilities arise here as well. As in the case of cancer treatment, an important consideration is the expression of retinoid receptors. After spinal cord injury the induction of RAR*β* appears to be a requirement. In addition to the classical transcriptional effects, retinoids and PPAR ligands can act via receptor-independent mechanisms. This, however, is a largely uncharted territory where therapeutic benefits remain to be discovered.

## Figures and Tables

**Table 1:** Anti-inflammatory effects of PPAR*α* and PPAR*γ* agonists on the molecular level.

Experimental system	Nuclear receptor	Regulated signals	Reference

Cytokine- and LPS-stimulated microglia	PPAR*α*, RXR	NO synthesis, IL-1*β*, IL-6, IL-12p40, TNF*α*, MCP-1	[78]
Activated macrophages	PPAR*α*, *γ*	iNOS, heme ogygenase (COX-2, HSP70 not affected)	[70]
Activated macrophages	PPAR*γ*	iNOS, MMP-9, scavenger receptor A	[80]
Activated human monocytes	PPAR*γ*	IL-1*β*, IL-6, TNF*α*	[81]
Activated macrophages	PPAR*γ*	Reactive oxigen species	[77]
LPS-stimulated microglia	PPAR*γ*	IL-1*β*, IL-6, TNF*α*, MCP-1, iNOS	[87]
LPS-stimulated microglia	PPAR*γ*	IL-6, TNF*α*, iNOS, COX-2	[85]
A*β*-stimulated microglia	PPAR*γ*	IL-6, TNF*α*, COX-2	[82]
LPS-stimulated astrocytes	PPAR*α*, RXR	IL-1*β*, IL-6, TNF*α*, iNOS, MCP-1	[88]
LPS-stimulated astrocytes	PPAR*γ*	IL-6, TNF*α*, iNOS, COX-2	[85]
LPS-stimulated astrocytes	PPAR*γ*	IL-1*β*, IL-6, TNF*α*, MCP-1, iNOS	[87]
Cerebellar injection of IFN*γ*, LPS	PPAR*γ*	iNOS, cell death	[89]
MPTP-treated mice	PPAR*γ*	iNOS, I*κ*B, cell death	[86]
APPV717I transgenic mice	PPAR*γ*	iNOS, COX-2, *β*-amyloid	[83]
Rat spinal cord injury	PPAR*γ*	IRF-1, IL-1*β*, IL-6, MCP-1, ICAM, Egr-1	[106]
Rat model for stroke	PPAR*γ*	IL-1*β*, iNOS, COX-2	[107]

## ABBREVIATIONS

A*β*: Beta amyloid peptide
ADH: Alcohol dehydrogenase
AP: Activating protein
BDA: Biotinylated dextran amine
BDNF: Brain-derived neurotrophic factor
CNS: Central nervous system
CNTF: Ciliary neurotrophic factor
COX: Cyclooxygenase
CRABP: Cellular retinoic acid binding protein
CRBP: Cellular retinol binding protein
dpo: *dies post operationem*
EGF: Epidermal growth factor
Egr: EGF receptor
ErbB: Tyrosine kinase related to the EGF recaptor
GAP43: Growth associated protein 43
ICAM: Intercellular cell adhesion molecule
IFN: Interferon, IL—interleukin
iNOS: Inducible NO synthase
IRF: Interferon regulatory factor
LPS: Lipopolysaccharide
LTB_4_: Leukotriene B4
MCP: Macrophage chemoattractant protein
MIP: Macrophage inflammatory protein
MMP: Matrix metalloproteinase
MPTP: 1-methyl-4-phenyl-1,2,3,6- tetrahydropyridine
NF*κ*B: Nuclear factor *κ*B NGF Nerve growth factor
NR(plus number): Nuclear receptor
PEA: Palmitoyl ethanolamide
15d-PGJ2: 15-deoxy-delta prostaglandin J2
PI3-K: Phosphoinositide 3-kinase
PNS: Peripheral nervous system
PPAR: Peroxisome proliferator-activated receptor
PPRE: PPAR response element
RA: Retinoic acid
RALDH: Retinaldehyde dehydrogenase
RAR: Retinoic acid receptor
RARE: Retinoic acid response element
RXR: Retinoid X receptor
SCI: Spinal cord injury
STAT: Signal transducer and activator of transcription
TGF: Transforming growth factor
TNF: Tumor necrosis factor
TR: Thyroid hormone receptor
TTNPB: 4-[(E)-2-(5,6,7,8-tetrahydro- 5,5,8,8-tetramethyl-2- naphthalenyl)-1-propenyl] benzoic acid
2,4-TZD: 2,4-thiazolidinedione
VCAM: Vascular cell adhesion molecule
